# Discovery of a Diverse Set of Bacteria That Build Their Cell Walls without the Canonical Peptidoglycan Polymerase aPBP

**DOI:** 10.1128/mBio.01342-21

**Published:** 2021-07-27

**Authors:** Sharanjeet Atwal, Suthida Chuenklin, Edward M. Bonder, Juan Flores, Joseph J. Gillespie, Timothy P. Driscoll, Jeanne Salje

**Affiliations:** a Public Health Research Institute, Rutgers University, Newark, New Jersey, USA; b Department of Biological Sciences, Rutgers University, Newark, New Jersey, USA; c Department of Microbiology and Immunology, School of Medicine, University of Maryland, Baltimore, Maryland, USA; d Department of Biology, West Virginia University, Morgantown, West Virginia, USA; e Mahidol-Oxford Tropical Medicine Research Unit, Faculty of Tropical Medicine, Mahidol University, Bangkok, Thailand; f Centre for Tropical Medicine and Global Health, Nuffield Department of Medicine, University of Oxford, Oxford, United Kingdom; Max Planck Institute for Terrestrial Microbiology

**Keywords:** peptidoglycan, penicillin binding proteins, *Rickettsiales*, obligate intracellular bacteria, endosymbionts, orthogonal chemical probes, Gram-negative bacteria, host-pathogen interactions

## Abstract

Peptidoglycan (PG) is a highly cross-linked peptide-glycan mesh that confers structural rigidity and shape to most bacterial cells. Polymerization of new PG is usually achieved by the concerted activity of two membrane-bound machineries, class-A penicillin binding proteins (aPBPs) and class-B penicillin binding proteins (bPBPs) in complex with shape, elongation, division, and sporulation (SEDS) proteins. Here, we have identified four phylogenetically distinct groups of bacteria that lack any identifiable aPBPs. We performed experiments on a panel of species within one of these groups, the *Rickettsiales*, and found that bacteria lacking aPBPs build a PG-like cell wall with minimal abundance and rigidity relative to cell walls of aPBP-containing bacteria. This reduced cell wall may have evolved to minimize the activation of host responses to pathogens and endosymbionts while retaining the minimal PG-biosynthesis machinery required for cell elongation and division. We term these “peptidoglycan-intermediate” bacteria, a cohort of host-associated species that includes some human pathogens.

## INTRODUCTION

The peptidoglycan (PG) cell wall is the load-bearing structure of most bacterial cells, conferring stiffness and osmotic protection. It comprises a mesh-like sacculus that usually covers the entirety of the cell membrane and is composed of long glycan strands cross-linked by short peptide side chains (1, 2). In Gram-positive bacteria this can be many layers thick, while in Gram-negative bacteria it is much thinner and surrounded by a second lipid membrane. As a highly conserved and essential constituent of almost all bacterial cells, PG is recognized by the eukaryotic immune system as a pathogen-associated molecular pattern and is a potent activator of innate immunity.

Polymerization of the PG precursor, lipid II, into growing PG strands has two enzymatic requirements: glycosyltransferase (GTase) activity to form β1-4-glycosidic bonds between disaccharide residues, and transpeptidase (TPase) activity to cross-link the peptide side chains on adjacent strands. Class A penicillin binding proteins (aPBPs) are large transmembrane proteins that possess both activities (3, 4). In contrast, class B PBPs (bPBPs) are transmembrane proteins that only possess TPase activity. It was recently shown that shape, elongation, division, and sporulation (SEDS) proteins, which form a complex with bPBPs, possess GTase activity in some organisms (5–9). Thus, it is possible that bPBP/SEDS and aPBPs form two complementary cell wall synthetic motors that both possess full TPase and GTase activity.The aPBPs are widespread and essential in most bacterial species and were long thought to be the primary drivers of PG polymerization; however, several lines of recent evidence suggest that most nascent PG growth is, in fact, driven by the newly described bPBP/SEDS activity, with aPBPs playing a supportive role in repairing cell wall defects. First, the subcellular localization of aPBP within the cytoplasmic membrane is distinct from foci formed by bPBP/SEDS pairs in the Gram-positive bacterium Bacillus subtilis and the Gram-negative bacterium Escherichia coli, indicating distinct activities (10). Second, it has been shown that all four aPBP genes can be deleted in B. subtilis to generate slow-growing but viable bacteria (11). Third, decreased expression of aPBPs in E. coli results in cells that remain rod shaped, but with decreased levels of PG cross-linking and less ability to sustain cell wall damage (12). These data suggest distinct, though partly complementary, roles for aPBPs and bPBPs in PG polymerization.

Here, we identify a group of bacteria that lack any identifiable aPBP genes but can synthesize a PG-like structure that is required for viability, demonstrating that aPBPs are dispensable for PG polymerization. Compared with closely related species that have aPBPs, the PG in these “PG-intermediate” (PGi) organisms is less abundant, and the cells do not have a uniform rod shape. These PGi organisms all exhibit an obligate intracellular or endosymbiotic lifestyle, and the osmotic protection and avoidance of innate immune receptors may have driven the selection for a minimal PG cell wall.

## RESULTS

### The class A penicillin binding protein gene has been lost at least four times during evolution.

**FIG 1:**
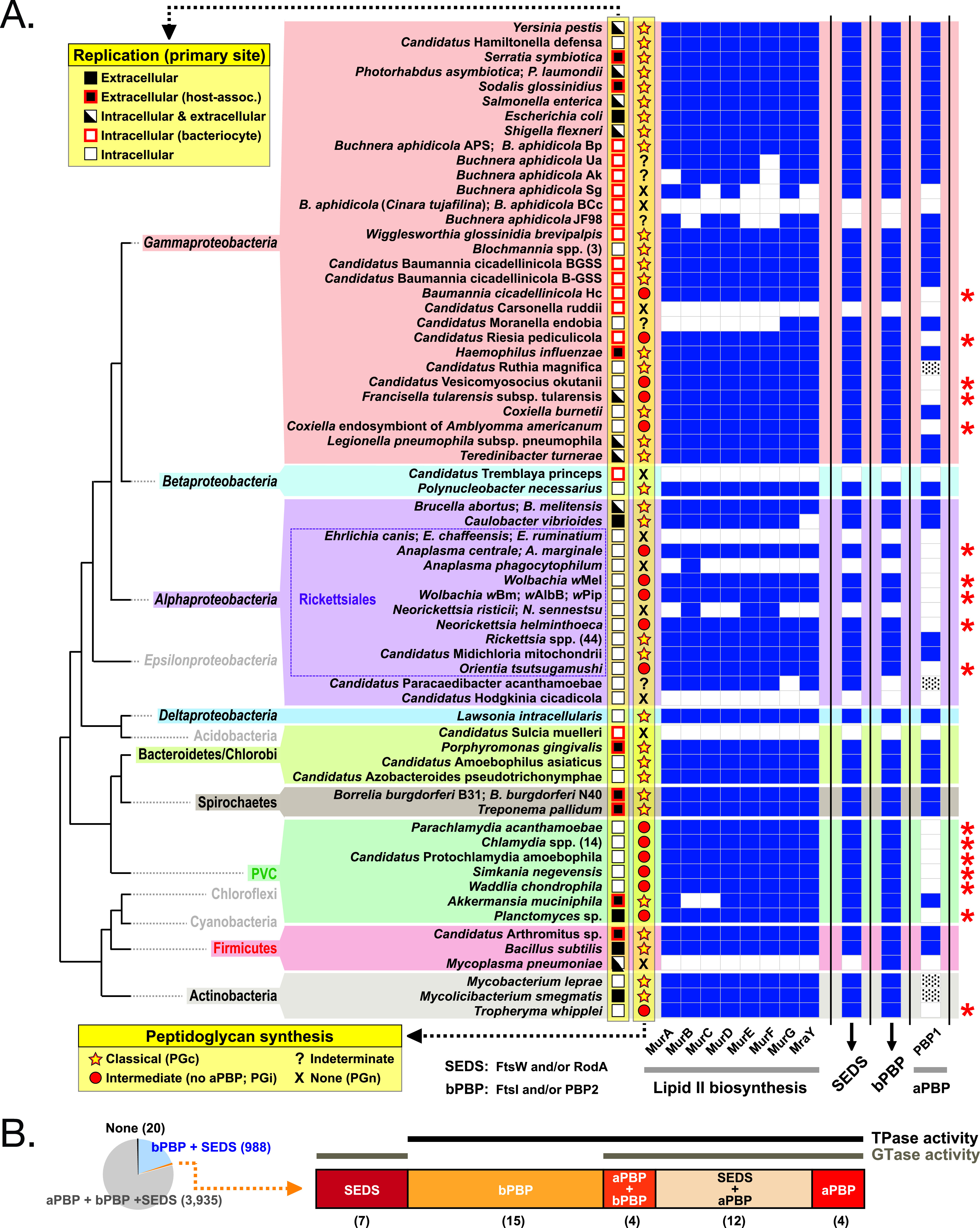
The class A PBP gene has been lost at least four times during evolution. (A) Analysis of key genes in the peptidoglycan biosynthesis pathway showing their presence or absence in obligate intracellular, facultative intracellular, host-associated, and free-living bacteria across 9 major bacterial groups. The predicted peptidoglycan (PG) status is shown as well as the primary site of replication of the bacteria. Genes involved in lipid II biosynthesis, SEDS family, class B PBPs, and class A PBPs were analyzed. The presence of a gene is shown by block color, the absence in white, and genes identified manually (not annotated as aPBP in KEGG) are shown with dashes. Bacterial organisms in the *Gammaproteobacteria* (*Baumannia cicadellinicola* Hc, “*Candidatus* Vesicomyosocius okutanii,” Francisella tularensis subsp. *tularensis*, *Coxiella* endosymbiont of *Amblyomma americanum*), *Alphaproteobacteria* (*Anaplasma* spp., *Wolbachia* spp., Orientia tsutsugamushi), PVC (Parachlamydia acanthamoebae, Chlamydia spp., “*Candidatus* Protochlamydia amoebophila,” Simkania negevensis, Waddlia chondrophila, *Planctomyces* sp.), and *Actinobacteria* (Tropheryma whipplei) all lack class A PBP homologs and are predicted to build intermediate PG-like structures (PGi) as highlighted by a red asterisk. PVC, *Planctomycetes*, *Verrucomicrobia*, and *Chlamydiae*; SEDS, shape, elongation, division, and sporulation; PBP, penicillin binding protein. Details of SEDS and bPBPs are shown in Fig. S1. (B) Relative quantification of bacterial strains containing different combinations of aPBP, bPBP, and SEDS proteins. This analysis was carried out on the 4,985 closed bacterial genomes available on KEGG in February 2020. Most bacteria (*n* = 4,923) either harbor all PG polymerization machines (80%) or qualify as PGi species (20%); a minority of species with other distributions (*n* = 42) are shown at the right as follows: SEDS only (*n* = 7), bPBP only (*n* = 15), bPBP and aPBP (*n* = 4), SEDS and aPBP (*n* = 12), and aPBP only (*n* = 4). Numbers of species in each category are shown in brackets. Bacteria binned to each category are listed in Table S1.

We previously used a comparative genomics approach to characterize the distribution of cell wall biosynthesis genes in obligate intracellular bacteria, which identified species that possessed almost all genes in the classical PG biosynthesis pathway but specifically lacked any identifiable aPBPs (13). Meeske et al. also performed a phylogenetic analysis showing that SEDS/bPBPs are more widely conserved than aPBPs and that some organisms lack aPBPs but retain a SEDS/bPBP synthase (6). Here, we extended this analysis to include 119 different strains across 9 major bacterial groups, including the identifiable obligate intracellular bacteria, facultative intracellular bacteria, and endosymbionts with complete genomes available in KEGG, as well as related free-living bacteria (Fig. 1A and Fig. S1). We identified clusters of bacteria that lack aPBPs in four unrelated groups, the *Rickettsiales*, the *Actinobacteria*, the *Gammaproteobacteria*, and the *Planctomycetes Verrucomicrobia Chlamydiae* (PVC) superphylum. These organisms were classified as PGi, with the criterion that they possess most lipid II biosynthesis genes, at least one SEDS and bPBP gene, but no identifiable aPBP gene. We also identified bacteria in five diverse groups that had lost the majority of their PG biosynthesis genes and would be predicted to lack PG synthesis capability (PG-negative [PGn]). In contrast, we term species that encode aPBPs, bPBPs/SEDS, and other genes in the PG biosynthesis “PG-classical” (PGc).

10.1128/mBio.01342-21.1 FIG S1 The class A PBP gene has been lost at least four times during evolution (extended version). Analysis of key genes in the peptidoglycan biosynthesis pathway showing their presence or absence in obligate intracellular, facultative intracellular, host-associated, and free-living bacteria across 9 phyla. The predicted peptidoglycan (PG) status is shown as well as the primary site of replication of the bacteria. Genes involved in lipid II biosynthesis, SEDS family, class B PBPs, and class A PBPs were analyzed. The presence of a gene is shown by block color, the absence in white, and genes identified manually (not annotated as aPBP in KEGG) are shown with dashes. Bacterial organisms in *Gammaproteobacteria* (*Baumannia cicadellinicola* Hc, “*Candidatus* Vesicomyosocius okutanii,” Francisella tularensis subsp. *tularensis*, *Coxiella* endosymbiont of *Amblyomma americanum*), *Alphaproteobacteria* (*Anaplasma* spp., *Wolbachia* spp., Orientia tsutsugamushi), PVC (Parachlamydia acanthamoebae, Chlamydia spp., “*Candidatus* Protochlamydia amoebophila,” Simkania negevensis, Waddlia chondrophila, *Planctomyces* sp.), and *Actinobacteria* (Tropheryma whipplei) all lack class A PBP homologs and are predicted to build intermediate PG-like structures (PGi). PVC, *Planctomycetes*, *Verrucomicrobia*, and *Chlamydiae*; SEDS, shape, elongation, division, and sporulation; PBP, peptidoglycan binding protein. Download FIG S1, PDF file, 0.1 MB. Copyright © 2021 Atwal et al. 2021 Atwal et al. https://creativecommons.org/licenses/by/4.0/ This content is distributed under the terms of the Creative Commons Attribution 4.0 International license.

10.1128/mBio.01342-21.3 TABLE S1 KEGGerator output indicating presence (1) or absence (0) of each KEGG ortholog involved in peptidoglycan synthesis, across all 4,989 bacterial genomes in KEGG. Each row contains a single taxon, including the KEGG group, genus, and KEGG organism identifier. Each column is a distinct ortholog identified with the KEGG identifier and, where applicable, gene locus. Columns Z, AA, and AB sum the number of different orthologs within the SEDS, bPBP, and aPBP families, respectively. Each sheet represents a different subset of bacteria. (A) All (*n* = 4,989) bacteria. (B) Bacteria (*n* = 3,935) containing at least one ortholog from all three families (SEDS, aPBP, bPBP). (C) Bacteria (*n* = 987) containing SEDS and bPBP but no aPBP. (D) Bacteria (*n* = 20) lacking SEDS, aPBP, and bPBP. (E) Bacteria (*n* = 7) containing SEDS but not aPBP or bPBP. (F) Bacteria (*n* = 19) containing bPBP but not SEDS or aPBP. (G) Bacteria (*n* = 4) containing aPBP and bPBP but not SEDS. (H) Bacteria (*n* = 12) containing SEDS and aPBP but not bPBP. (I) Bacteria (*n* = 4) containing aPBP but not SEDS or bPBP. Download Table S1, XLSX file, 1.0 MB. Copyright © 2021 Atwal et al. 2021 Atwal et al. https://creativecommons.org/licenses/by/4.0/ This content is distributed under the terms of the Creative Commons Attribution 4.0 International license.

10.1128/mBio.01342-21.4 TABLE S2 Taxonomic groups included in the current study. Each entry is identified by its KEGG organism identifier, genus, taxon name, and lifestyle (extracellular, extracellular-attached, intracellular-obligate, intracellular-facultative, bacteriocytic, bacteriocytic; extracellular, or free-living). (A) All 117 taxa in the analysis. (B) All taxa binned into 65 groups, based on taxonomic relatedness and PGP profile similarity. Download Table S2, XLSX file, 0.01 MB. Copyright © 2021 Atwal et al. 2021 Atwal et al. https://creativecommons.org/licenses/by/4.0/ This content is distributed under the terms of the Creative Commons Attribution 4.0 International license.

10.1128/mBio.01342-21.5 TABLE S3 KEGG identifiers for all orthologs included in the current study, grouped by broad biochemical pathway. Download Table S3, XLSX file, 0.01 MB. Copyright © 2021 Atwal et al. 2021 Atwal et al. https://creativecommons.org/licenses/by/4.0/ This content is distributed under the terms of the Creative Commons Attribution 4.0 International license.

This result showed that aPBPs are dispensable for growth in host-associated bacteria. We extended this analysis to all 4,985 closed bacterial genomes available on KEGG without distinguishing organisms by lifestyle (Fig. 1B and Table S1). We found that the majority (3,935) encoded aPBPs, bPBPs, and SEDS (PGc), a smaller fraction (988) had specifically lost aPBPs (PGi), and a small number (20) had lost all three classes of genes (PGn). In contrast, we only identified 20 instances of strains that had retained an aPBP but lost SEDS and/or bPBP. This analysis builds on previous reports of organisms lacking aPBPs (6, 13) and demonstrates that while aPBPs are dispensable for growth, SEDS/bPBPs have been almost universally retained.

The *Rickettsiales* are an order of obligate intracellular bacteria that are dominated in nature by arthropod endosymbionts (*Wolbachia* strains and other species/strains) but also include a number of important arthropod-transmitted animal pathogens (species of *Neorickettsia*, *Anaplasma*, *Ehrlichia*, *Orientia*, *Rickettsia*). The pathogens *Ehrlichia* spp., Anaplasma phagocytophilum, Neorickettsia sennetsu, and Neorickettsia risticii were all classified as PGn, while the closely related pathogens Anaplasma marginale, Anaplasma centrale, Neorickettsia helminthoeca, Orientia tsutsugamushi, and *Wolbachia* strains were all classified as PGi. Remarkably, despite significant genome reduction, all 44 *Rickettsia* species/strains contain a full complement of PG synthesis genes and were classified as PGc.

The *Gammaproteobacteria* include obligate intracellular, bacteriocyte-associated intracellular, facultative intracellular, host-associated extracellular, and free-living extracellular species. Out of 30 *Gammaproteobacteria* strains, we identified 7 PGn/unclassified, 6 PGi and 17 PGc. We found three instances where closely related strains had distinct classifications: (i) Buchnera aphidicola, where five strains were predicted to be PGn (or unclassified) and two other strains of *B. aphidicola* (APS, Bp) were predicted to be PGc. *B. aphidicola* strain JF98 was the only strain in our data set that retained aPBP but lost bPBP/SEDS; however, this also lacks *murB*, *murE*, and *murF* and is likely in the process of pathway degradation. (ii) Baumannia cicadellinicola, where one strain (“*Candidatus* Baumannia cicadellinicola” BGSS) was classified as PGc while another strain (*B. cicadellinicola* Hc) lacked aPBP and was classified as PGi. (iii) *Coxielliae*, where Coxiella burnettii was classified as PGc and a *Coxiella* endosymbiont of Amblyomma americanum was classified as PGi.

The PVC group includes obligate intracellular pathogens (species of Chlamydia, *Waddlia*), amoeba endosymbionts (*Simkania* spp.), and extracellular bacterial species (Akkermansia muciniphila, *Planctomyces* spp.). We analyzed seven distinct species, and all except *Akkermansia* were classified as PGi. *Akkermansia*, which is a host-associated extracellular bacterium, retained aPBP and was classified as PGc.

The *Actinobacteria* are a group of Gram-positive bacteria that includes Mycobacterium species. We analyzed three species and found that Tropheryma whipplei, which is a human pathogen generally thought to have an obligate intracellular lifestyle, was classified as PGi, while the other species were classified as PGc.

Together, this analysis led us to the hypothesis that the PGi bacteria produce a PG wall that is different from that produced by PGc bacteria and that this would confer an advantage within the obligate intracellular/endosymbiotic life cycle. In order to test this hypothesis, we selected a group of six related organisms from within one of these groups, the *Rickettsiales*, and set out to determine whether they synthesized PG walls with different characteristics. For this analysis we selected representative PGc (Rickettsia canadensis), PGi (Orientia tsutsugamushi, *Wolbachia* endosymbiont of *melanogaster* [Wm], and Anaplasma marginale), and PGn (Anaplasma phagocytophilum, and Ehrlichia chaffeensis) species.

### PG-intermediate bacterial species build a PG-like structure.

**FIG 2:**
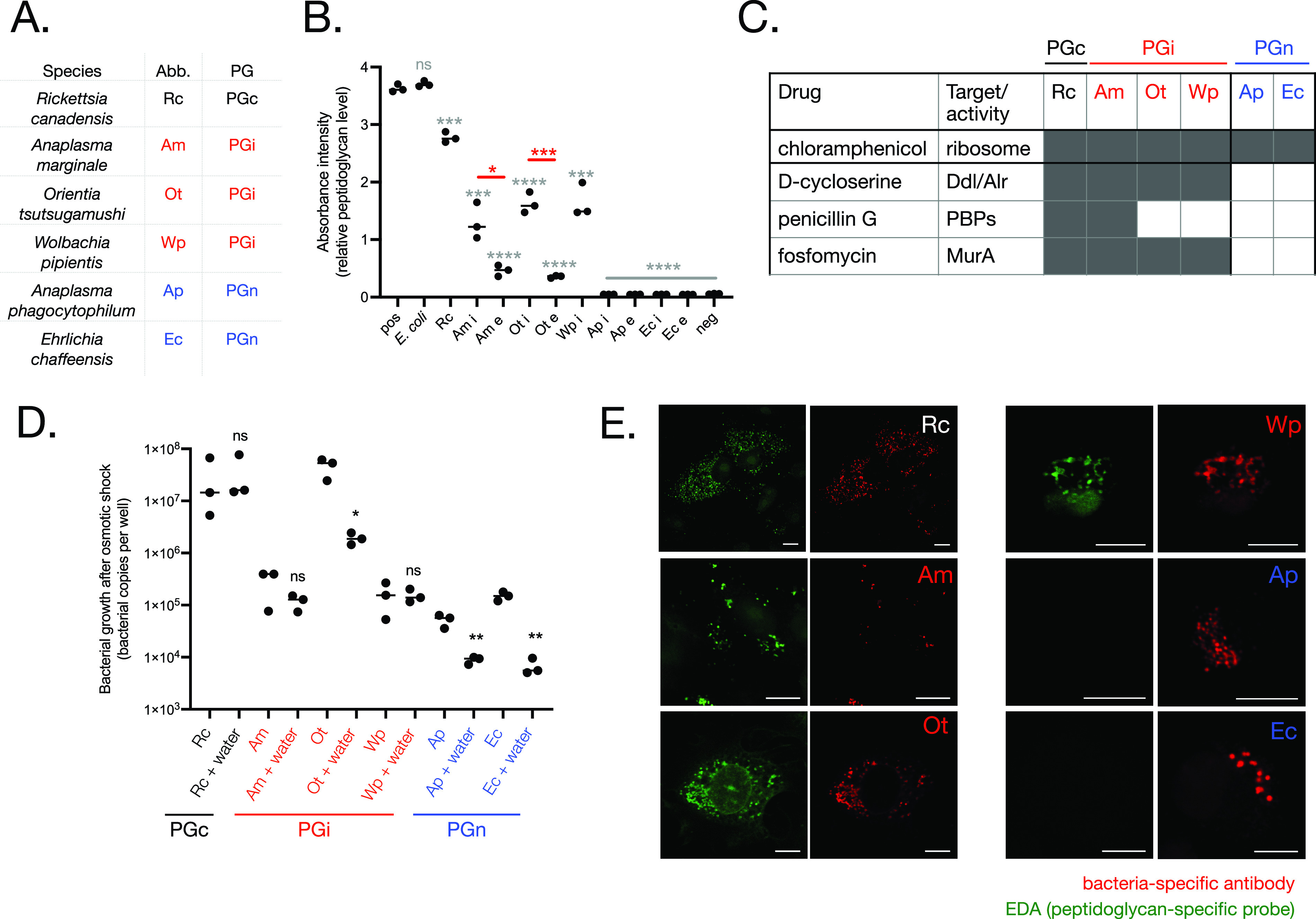
PG-intermediate *Rickettsiales* species build a PG-like structure that confers osmotic protection. (A) Overview of species in this figure. (B) Quantification of relative PG levels using a NOD1 reporter cell line. Extracellular (e) and intracellular (i) bacteria were isolated from host cells and quantified by qPCR, and 1 × 10E4 bacteria were added to HEK-Blue NOD1 reporter cells. Activation of NOD1 in response to peptidoglycan was measured by secreted alkaline phosphatase activity. Positive control (pos) was 10 μg/ml iE-DAP. Individual values and median are shown, and statistical analyses show the difference between two groups as measured by an unpaired *t* test using the software GraphPad Prism. Gray asterisks show the results of statistical comparisons between each group and the positive control. Red asterisks show the difference between the i and e population of a particular bacterial species. (C) Sensitivity of *Rickettsiales* to cell wall-targeting drugs. Bacteria were grown in the presence of drugs at the following concentrations: chloramphenicol, 100 μg/ml; penicillin G, 150 μg/ml; d-cycloserine 250 μg/ml; and phosphomycin, 40 μg/ml. The bacterial copy number after growth was compared with that in the absence of drugs. A bacterial species was scored as susceptible to that drug if the bacterial copy number was reduced in a statistically significant manner as measured by an unpaired *t* test. Gray cell, bacteria susceptible to drug; white cell, bacteria resistant to drug. Raw data are given in Fig. S1. (D) Quantification of bacterial regrowth after 10 min of incubation in sucrose phosphate buffer (SPG) or water. Bacteria were isolated from host cells, exposed to osmotic shock (pure water) or an osmotically protective buffer (SPG) for 10 min, and then grown in host cells for 7 days. Bacterial growth was measured by qPCR. Individual values and median are shown, and an unpaired *t* test was used to compare the water-treated and untreated groups within each bacterial species using the software GraphPad Prism. (E) Visualization of a PG-like structure in R. canadensis and PGi species. Bacteria were grown in the presence of a d-alanine analog containing an alkyne group (EDA). After fixation, incorporation of probes into nascent PG was detected by labeling with azide-alexa488 using a copper catalyzed click reaction. Bacteria were counterstained using bacterium-specific antibodies. PGc (R. canadensis) and PGi (A. marginale/Wp/O. tsutsugamushi) species could be labeled with EDA, while PGn (E. chaffeensis/A. phagocytophilum) species could not. Throughout the figure, PGc (R. canadensis) is indicated in black, PGi (A. marginale, O. tsutsugamushi, Wp) in red, and PGn (A. phagocytophilum, E. chaffeensis) in blue. *P* values throughout: *, *P* ≤ 0.05; **, *P* ≤ 0.01; ***, *P* ≤ 0.001; ****, *P* ≤ 0.0001.

We used a NOD1 reporter assay to assess whether the PGc and PGi species generated detectable PG-like structures. NOD1 is a mammalian innate immune receptor that detects PG fragments containing the PG-specific amino acid *meso-*DAP that is commonly found in Gram-negative bacterial PG (14). The NOD1 reporter assay measures the response of human embryonic kidney (Hek1) reporter cells to *meso-*DAP-containing PG fragments (15). We purified 6 *Rickettsiales* species from host cells and added them to NOD1 reporter cells at a constant bacterial concentration (Fig. 2A and B). While PGn species did not stimulate a NOD1 response, both PGc and PGi induced NOD1 activation. This demonstrated the presence of *meso-*DAP-containing PG in R. canadensis, A. marginale, O. tsutsugamushi, and *Wolbachia pipientis* (Wp). The magnitude of this activation was higher in the PGc species R. canadensis than in PGi organisms, suggesting a higher abundance of PG in this species.

We analyzed PG levels at different stages of the bacterial life cycle. The *Anaplasmataceae* A. marginale, A. phagocytophilum, and E. chaffeensis undergo a biphasic life cycle in which they differentiate between a replicative intracellular form and an infectious extracellular form (16). We recently showed that O. tsutsugamushi also differentiates into distinct intracellular and extracellular populations (manuscript in preparation). In contrast, differentiation has not been described in R. canadensis and Wp. We isolated bacteria from intracellular and extracellular populations in A. marginale, O. tsutsugamushi, A. phagocytophilum, and E. chaffeensis and found that in the PGi species A. marginale and O. tsutsugamushi, the PG levels were significantly higher in the intracellular population (Fig. 2B), at which time the bacteria are actively undergoing growth and division. PG could not be detected in either intracellular or extracellular populations of A. phagocytophilum or E. chaffeensis.

Next, we determined the susceptibility of the same six species to PG-targeting drugs, in order to determine whether they made a PG-like structure that was required for their growth (Fig. 2C and Fig. S2). We used the bacterial ribosomal inhibitor chloramphenicol as a positive control, because this is known to be effective against *Rickettsiales*. We tested the susceptibility to d-cycloserine (targets proteins involved in the isomerization of l-Ala to d-Ala and its dimerization into d-Ala-d-Ala); penicillin G (targets TPase activity of aPBP and bPBP), and fosfomycin (targets MurA, a cytoplasmic protein required for early stages of synthesis of the PG precursor lipid II). The PGc species R. canadensis was sensitive to all cell wall-targeting drugs that we tested. The PGi organisms were all sensitive to d-cycloserine and fosfomycin, consistent with the presence of an essential PG-like structure and as shown previously for some of these (17–19). A. marginale was susceptible to penicillin G; both O. tsutsugamushi and Wp were not. The susceptibility of PGi organisms to d-cycloserine and fosfomycin demonstrates that they build a PG-like structure that is required for their growth; however, the differential susceptibility of PGi organisms to penicillin G cannot currently be explained and may reflect different PBP modifications or differences in membrane structure or permeability. PGn organisms were insensitive to all the cell wall-targeting drugs that we tested, consistent with a lack of a functional PG cell wall.

10.1128/mBio.01342-21.2 FIG S2 Drug sensitivity assays. Bacterial copy number after 5 days (7 days for *Wolbachia)* of growth in the presence or absence of drugs was quantified by qPCR. Growth in the presence of a drug was normalized by growth in the absence of drug within the same experiment. RC, Rickettsia canadensis, grown for 5 days in Vero cells; AM, Anaplasma marginale grown for 5 days in Vero cells; OT, Orientia tsutsugamushi grown for 5 days in L929 cells; WP, Wolbachia pipientis grown for 7 days in JW18 cells; EC, Ehrlichia chaffeensis grown for 5 days in DH82 cells; AP, Anaplasma phagocytophilum grown for 5 days in HL60 cells. Drug concentrations were 100 μg/ml chloramphenicol, 150 μg/ml penicillin G, 250 μg/ml d-cycloserine, and 40 μg/ml phosphomycin. Statistical analysis between the untreated and drug-treated groups was carried out using an unpaired *t* test with GraphPad Prism software. Individual values and median are shown. *P* values: *, *P* ≤ 0.05; **, *P* ≤ 0.01; ***, *P* ≤ 0.001. Download FIG S2, PDF file, 0.1 MB. Copyright © 2021 Atwal et al. 2021 Atwal et al. https://creativecommons.org/licenses/by/4.0/ This content is distributed under the terms of the Creative Commons Attribution 4.0 International license.

Next, we tested whether the PG-like structure of PGi species conferred osmotic protection. We isolated bacteria from their host cells, exposed them to hypoosmotic shock (water), and assessed the effect on subsequent bacterial growth (Fig. 2D). We found that the PGn species were highly sensitive to hypoosmotic shock, whereas growth of the PGi species A. marginale and Wp as well as the PGc R. canadensis was not affected. Growth of the PGi O. tsutsugamushi was affected by hypoosmotic shock, demonstrating that the amount of PG in this organism was not sufficient to confer complete protection. While the mechanistic basis of this difference is unknown, it may reflect the fact that A. marginale and Wp replicate within a membrane-bound vacuole and may have evolved additional rigidity in their membranes to survive vacuole-associated hypoosmolarity compared with the cytoplasm-residing PGi species O. tsutsugamushi.

### Visualization of a PG-like structure in PG-intermediate species using a PG-specific metabolic probe.

We used a clickable d-amino acid analog, ethynyl-d-alanine (EDA), to determine the spatial localization of PG in PGc and PGi *Rickettsiales* species (Fig. 2E). This is an orthogonal chemical probe that incorporates into the PG of growing bacterial cells and can be conjugated to a fluorophore after fixation for fluorescence microscopy analysis. We have previously used this to label the PGi O. tsutsugamushi (17). This probe labeled both PGc and PGi species in our study, indicating the presence of some form of PG sacculus. However, we found that the labeling was frequently unsuccessful despite carefully controlling for variability. This may be due to batch-dependent differences in permeability of host cells or differences in the activity of PG synthesis machinery at different stages of bacterial growth. The PGn species A. phagocytophilum and E. chaffeensis could never be labeled with EDA, consistent with our hypothesis that these lack any PG-like structure and demonstrating that the probes do not bind nonspecifically to bacterial cells under these experimental conditions. It has been shown that some PGi *Chlamydiales* species only synthesize PG at their septum (20). Such localization was never observed in any of the species studied here.

### Analysis of the cell shape of PGc, PGi, and PGn *Rickettsiales*.

**FIG 3:**
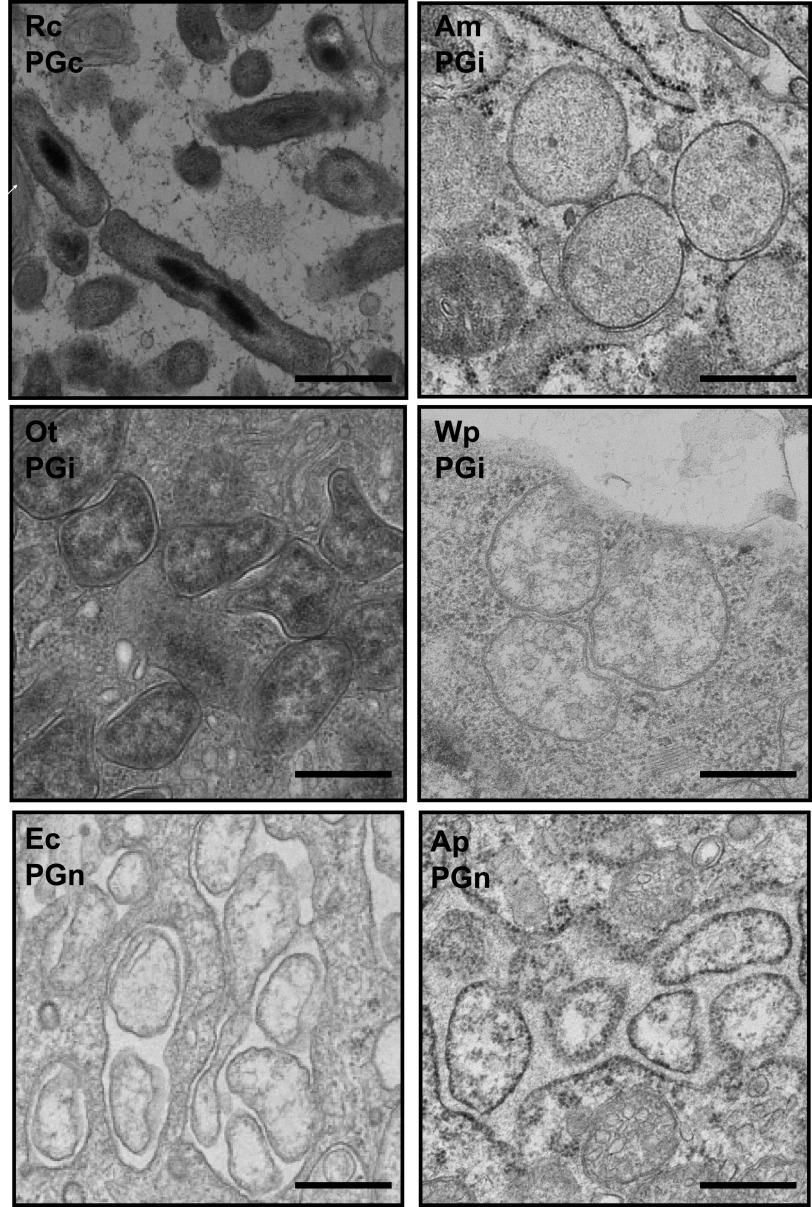
Transmission electron microscopy analysis of PGc, PGi, and PGn *Rickettsiales*. Bacteria were grown in host cells and prepared for fixed thin-section transmission electron microscopy. Micrographs show that the PGi R. canadensis is rod-shaped compared with PGi and PGn *Rickettsiales*. PGi and PGn bacteria are round and/or pleiomorphic, reflecting the low level or absence of PG in their cell walls.

The low levels of NOD1 activation in PGi species (Fig. 2A), combined with the fact that PG has been difficult to detect in both O. tsutsugamushi and Wp, suggests that the absence of aPBP leads to a reduced amount of PG in the cell wall. We reasoned that this may result in reduced structural rigidity and that this would affect bacterial shape. Infected cells were examined by transmission electron microscopy to document the shape of the six *Rickettsiales* species within host cells (Fig. 3). We found that PGc R. canadensis forms regular, rod-shaped cells, while PGi and PGn species adopt irregular and/or round cells. This finding is consistent with an electron microscopy analysis showing that the *Gammaproteobacteria* “*Candidatus* Baumannia cicadellinicola” strain BGSS (endosymbiont of blue-green sharpshooter), classified here as PGc, is rod shaped while the closely related *Baumannia cicadellinicola* strain Hc (endosymbiont of glassy-winged sharpshooter) classified here as PGi, is irregular and non-rod shaped (21).

## DISCUSSION

Here, we used comparative genomics to identify 16 species across 4 major bacterial groups that have lost aPBPs but retained genes for synthesis of the PG precursor lipid II as well as at least one bPBP and one SEDS gene. We characterized three of these species from within the order *Rickettsiales* and showed that they generate a PG-like structure that is required for bacterial growth. It has already been shown that multiple chlamydial species, which are not closely related to *Rickettsiales*, generate minimal PG-like structures (20, 22), and we hypothesize that an absence of aPBPs in *Gammaproteobacteria* and *Actinobacteria* will also generate bacteria with similar cell wall characteristics.

The species that lack aPBP were almost all associated with an intracellular lifestyle (15 out of 16). There are two possible reasons why adaptation to an intracellular lifestyle would confer selective pressure to reduce the amount of PG in the bacterial cell wall. First, PG is a strong stimulator of the host immune system via peptidoglycan recognition proteins such as NOD1/NOD2 and PG recognition proteins in mammals and peptidoglycan recognition proteins (PGRPs) in invertebrates. Activation of these systems leads to induction of antibacterial mechanisms that intracellular bacteria aim to evade, and we show here that both PGc and PGi Rickettsiales are able to activate NOD1. There is substantial variation in the abundance and distribution of PGRPs in invertebrate hosts (23), and differences in the magnitude of PG-sensing between hosts may underpin why some host-associated bacteria were able to retain a classical cell wall (PGc) while others were under strong selective pressure to reduce or remove it (PGi/PGn). Second, the intracellular niche (whether cytosolic or vacuolar) offers an osmotically protective environment, and therefore the bacteria may not require cell walls with the same rigidity as their free-living counterparts. *Rickettsia* species counter this argument yet are the only analyzed *Rickettsiales* species that synthesize lipopolysaccharide (24), which may require a more rigid cell wall for scaffolding this large glycoconjugate.

**FIG 4:**
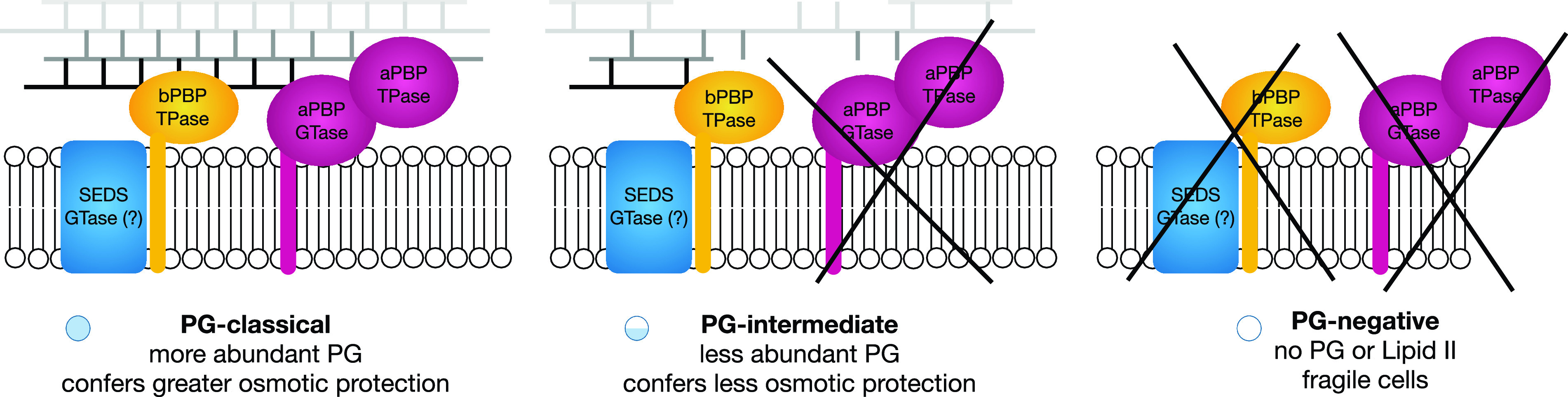
Predicted PG polymerization in PG-classic, PG-intermediate, and PG-negative organisms. SEDS, shape, elongation, division, and sporulation proteins (FtsW, RodA); bPBP, class B penicillin binding protein (PBP2, FtsI); aPBP, class A penicillin binding protein; TPase, transpeptidase activity; GTase, glycosyltransferase activity.

How do PGi bacteria build a PG wall in the absence of aPBPs, a bifunctional TPase and GTase normally considered a major driver of PG polymerization? All the PGi strains identified in our analysis retained at least one copy of the two known bPBP and SEDS genes. bPBPs have TPase activity, and SEDS from E. coli and B. subtilis are known to have GTase activity (5, 6, 8, 9). Thus, it is plausible that bPBP/SEDS complexes are the major drivers of PG polymerization in aPBP-negative PGi organisms. This is supported by the observation that aPBPs in B. subtilis are dispensable for growth (11), resulting in a reliance on bPBP/SEDS-driven PG polymerization analogous to the PGi species in our study. Our model for PG polymerization in PGc, PGi, and PGn bacteria is shown in Fig. 4.

These results also raise the following question: what is the structure of the bacterial cell wall in PGi organisms? The reduced level of NOD1 activation shown in this study compared with E. coli and the PGc R. canadensis, the difficulty in d-alanine orthogonal probe labeling, and the historical inability to detect PG in PGi organisms (18, 25–28) suggest that the PG in these bacteria is not abundant, and in organisms with biphasic lifestyles this structure is further depleted during some stages of the growth cycle. Since PG in Gram-negative bacteria is only one or a few layers thick (1), the observed low abundance is likely to result from PG consisting of shorter glycan strands and/or less peptide cross-linking. Diminished cross-linking within the PG sacculus would result in a larger mesh size and lower structural support to the underlying membrane (Fig. 4). This would explain the reduced osmotic protection of PGi *Rickettsiales* compared with the closely related PGc relatives, as well as the loss of regularity in cell shape that would result from a less extensive—and therefore less rigid—PG sacculus. This interpretation of the PG structure in PGi organisms is consistent with a role for aPBP in increasing the cross-linking and repairing damage (12) rather than being the major driver of PG polymerization.

Together, these data show that aPBP is dispensable for PG polymerization and raises questions about the structure and synthesis of PG in organisms that naturally lack this important component of the PG biosynthesis machinery.

## MATERIALS AND METHODS

### Comparative genomics analyses.

A total of 119 bacterial taxa (Table S2) were selected for comparative analysis based on their lifestyle, phylogenomic relationship, and genome status (closed genomes only). Then, 32 genes of interest were chosen (Table S3), 10 lipid II biosynthesis genes, 2 SEDS family protein genes, 13 bPBP genes, and 7 aPBP genes. Identifiers for all taxa and genes were located in the Kyoto Encyclopedia of Genes and Genomes (KEGG) and used as input to kegghole, part of the keggerator package (24). This software queries KEGG for the presence of the input genes in all input taxa and generates a presence/absence matrix. Genomes that appeared to lack an input gene according to KEGG were manually queried using the protein sequence from a related organism and NCBI’s blastp program (default settings except for an E value cutoff of 1). Genes absent from all 117 query taxa were removed. Finally, phylogenetically related taxa with identical patterns of gene presence/absence were compressed into taxon groups for simplicity.

### Bacterial propagation and quantification.

The cell lines and bacteria listed in Table 1 were used.

**TABLE 1:** Cell lines and bacteria used in the study

Bacterial species	Strain	Source of strain	Host cell type	Source of cell line
Rickettsia canadensis	CA410	Stuart Blacksell	L929 (mouse fibroblast)HeLa (human epithelial)	ATCC CCL-1ATCC CCL-2
Anaplasma marginale	291	ATCC (VR-1444)	Vero (monkey epithelial)	ATCC CRL-1586
Orientia tsutsugamushi	UT76	Kelly Brayton	Vero (monkey epithelial)	ATCC CRL-1586
Wolbachia pipientis	wMel	Irene Newton	JW18 (*Drosophila*)	Irene Newton
Anaplasma phagocytophilum	HGE1	Kelly Brayton	DH82 (dog macrophage)	ATCC CRL-10389
Ehrlichia chaffeensis	Arkansas	Jere Mcbride	DH82 (dog macrophage)	ATCC CRL-10389

### Cell culture.

L929 cells were grown in RPMI 1640 medium with HEPES (Thermo Fisher Scientific, USA; 22-400-071) supplemented with 10% heat-inactivated fetal bovine serum (FBS; Thermo Fisher Scientific; 16140071) in 25-cm^2^ flasks at 37°C and 5% CO_2_. HeLa cells were grown in 25-cm^2^ flasks with growth medium (Dulbecco’s modified Eagle’s medium [DMEM]; Thermo Fisher Scientific; 21013) supplemented with 10% heat-inactivated FBS (Thermo Fisher Scientific; 16140071) at 37°C and 5% CO_2._ Vero cells were grown in RPMI 1640 medium with HEPES, supplemented with 10% heat-inactivated FBS in 25-cm^2^ flasks at 37°C and 5% CO_2_. DH82 cells were grown in 25-cm^2^ flasks with Eagle’s minimum essential medium (EMEM; Sigma, USA; M0325) with 10% heat-inactivated FBS at 37°C and 5% CO_2_. JW18 cells were grown in Schneider’s insect medium (Sigma; S0146) with 10% heat-inactivated FBS at 25°C.

HEK-Blue hNOD1 (InvivoGen, USA; chkb-hnod1) cells were used for NOD1 reporter assays and were grown in 25-cm^2^ flasks at 37°C and 5% CO_2_ with growth medium DMEM with 4.5 g/liter glucose, 10% heat-inactivated FBS, 100 U/ml penicillin, 100 mg/ml streptomycin, 100 mg/ml Normocin, and 2 mM l-glutamine. Selective antibiotics, 30 μg/ml of blasticidin and 100 μg/ml of Zeocin, were added after passage 2 to maintain the cell line.

Bacterial copy number was determined by extracting DNA using alkaline lysis treatment and then performing quantitative PCR (qPCR) relative to known standards (29) (Table 2).

**Table 2:** Primers and probes used in the study

Primer/probe name	Target organism(s)	Target gene	Sequence
tsa47f	Orientia tsutsugamushi	*tsa47*	TCCAGAATTAAATGAGAATTTAGGAC
tsa47r			TTAGTAATTACATCTCCAGGAGCAA
tsa47probe			[6FAM]TTCCACATTGTGCTGCAGATCCTTC[TAM]
Rick16Sf	*Rickettsiaceae*: Orientia tsutsugamushi/ Rickettsia canadensis	*16S*	GCTACACGCGTGCTACAATGG
Rick16SRr			TGTGTACAAGGCCCGAGAACG
Rick16Sprobe			[6FAM] ATCGCTAGTAATCGCGGATCAGCATGCC [TAM]
Ana16Sf	*Anaplasmataceae*: *Wolbachia/Anaplasma/Ehrlichia*	*16S*	ACTGGAGGAAGGTGGGGATG
Ana16Sr			TGATCCACGATTACTAGCGATTCC
Ana16Sprobe			[6FAM] TGGGCTACACACGTGCTACAATGG [TAM]

### Quantification of relative peptidoglycan levels using NOD1 reporter assay.

HEK-Blue hNod1 cells (Invitrogen, USA; hkb-hnod1) were grown in DMEM growth medium with 4.5 g/liter glucose, 10% heat-inactivated fetal bovine serum, 100 U/ml penicillin, 100 mg/ml streptomycin, 100 mg/ml Normocin, and 2 mM l-glutamine plus selective antibiotics (30 μg/ml of blasticidin and 100 μg/ml of Zeocin). Cells were seeded on clear-bottom black 96-well plates (Corning, USA; 29444-008) 2 days before infection. Bacteria were taken from prepared aliquots kept at −80°C in sucrose phosphate glutamine (SPG). All bacteria were heat-inactivated at 90°C for 30 min before being added to host cells. All infections were carried out in triplicate. After 2 days of infection, growth medium was replaced with HEK-Blue detection medium (InvivoGen, USA; hb-det2) for secreted embryonic alkaline phosphatase (SEAP) detection. The plate was further incubated at 37°C and 5% CO_2_ and quantified by spectrophotometry (Synergy H1; BioTek) at 640 nm from 6 h of the addition of detection media. Results were added to Prism (GraphPad Software, San Diego, CA, USA), and an unpaired *t* test was performed to compare bacterial SEAP levels to positive-control iE-DAP (γ-d-Glu-mDAP) at 10 μg/ml, as well as to perform pairwise comparisons between intracellular and extracellular bacteria in the case of *Wolbachia* and Orientia tsutsugamushi species.

### Growth inhibition experiments.

Bacteria were grown in 12-well plates in their respective cell lines in the presence of drugs at the following concentrations: 100 μg/ml chloramphenicol, 150 μg/ml penicillin G, 250 μg/ml d-cycloserine, and 40 μg/ml phosphomycin. After 5 days of growth (7 days for *Wolbachia*) bacterial DNA was extracted and quantified by qPCR as described above. For quantifying the effect of osmotic shock, bacteria were isolated from host cells, resuspended in pure water for 10 min, and then grown in fresh host cells. Bacterial growth after 5 days was quantified by qPCR. Results were added to Prism (GraphPad Software, San Diego, CA, USA), and an unpaired *t* test was performed to compare growth in the presence or absence of drugs or growth in the presence or absence of osmotic shock.

### Immunofluorescence labeling, click labeling, and confocal microscopy.

All fixed cells were permeabilized in 0.5% Triton X for 30 min, 100% ethanol for 1 h on ice, and 1 mg/ml lysozyme in sterile tris-EDTA for 1 h at room temperature. Primary antibodies were added for 1 h at 37°C (TSA56, 13-6, ANAF16C1, FtsZ, dog serum, and anti-p44; see Table 3). Samples were washed 3 times with phosphate-buffered saline-bovine serum albumin (PBS-BSA), and then the appropriate secondary antibodies were diluted 1:500 and incubated for 30 min at 37°C in the dark (goat anti-rat IgG Alexafluor 555 conjugate [Thermo Fisher A-21434], goat anti-rabbit, Alex Fluor 594 [Thermo Fisher A-11012], goat anti-mouse IgG superclonal Alexa Fluor 555 [Thermo Fisher A28180], and goat anti-canine IgG Texas red [Novus Biologicals NBP173511]). The nuclear stain Hoechst was diluted to 1:1,000 and included with the secondary antibody incubation. Before addition of the mounting medium, cells were washed with 1× PBS again.

**Table 3:** Primary antibodies used in the study

Species	Antibody name	Source
O. tsutsugamushi	TSA56	In-house
*R. canadensis*	13-6	Ted Hackstadt, Rocky Mountain Laboratories
*R. canadensis*	4994	Ted Hackstadt, Rocky Mountain Laboratories
A. marginale	ANAF16C1 (Msp5)	Kelly Brayton, Washington State University
A. phagocytophilum	FtsZ	Jason Carlyon, Virginia Commonwealth University
Erlichia canis	Serum	Roman Ganta, Kansas State University
*Wolbachia*	Anti-p44	Irene Newton, Indiana University

The metabolic click-labeling is based on the Click-iT l-homopropargylglycine (HPG) Alexa Fluor protein synthesis assay kits (molecular probe by Life Technologies). To incorporate HPG at each time point, infected cells were incubated in the minimal medium without l-methionine (Dulbecco’s modified Eagle’s medium [DMEM], catalog [cat.] no. 21013) containing 25 μM HPG for 30 min at 37°C. Labeled bacteria were washed twice in PBS plus 1 mg/ml BSA before fixing with 1% formaldehyde or methanol (antibody 13-6 only) and subsequently permeabilized with 0.5% Triton X for 30 min, 100% ethanol for 1 h on ice, and 1 mg/ml lysozyme in sterile tris-EDTA for 1 h at room temperature. After washing with PBS plus 1 mg/ml BSA, Click-iT reaction cocktail was incubated with cells for 30 min at room temperature protected from light. The component of Click-iT reaction cocktail is based on Click-iT HPG Alexa Fluor protein synthesis assay kits [cat.] no. C10428. The azide dye (Alexa Fluor 488; Invitrogen A10266) was used at final concentration of 5 μM. After the click reaction, the cells were ready for immunofluorescent fluorescence labeling and imaging as described above.

Imaging was performed using an Observer Z1 LSM700 confocal microscope with an HBO 100 illuminating system equipped with a ×63/1.4 Plan-APOCHROMAT objective lens (Carl Zeiss, Germany) and 405-nm, 488-nm, and 555-nm laser lines. In some cases, we used a TCS SP8 confocal microscope (Leica Microsystems, Germany) equipped with a ×63/1.4 Plan-APOCHROMAT oil objective lens with a 1.4-mm working distance and 405-nm, 488-nm, 552- nm, and 638-nm laser lines.

### Transmission electron microscopy.

Bacteria were grown in their respective cell lines, harvested by trypsinization, and pelleted at 1,000 × *g* in a microcentrifuge. Culture medium supernatants were removed, and the resultant cell pellets were fixed using 2.5% formaldehyde, 2.5% glutaraldehyde, and 0.1 M sodium cacodylate buffer, pH 7.4 (EMS catalog no. 15949). After overnight fixation at 4°C, the pellets were gently rinsed and floated in 0.1 M sodium cacodylate buffer, pH 7.4 and postfixed with 1% osmium tetroxide in 0.1 M sodium cacodylate buffer, followed by en-bloc staining in 1% aqueous uranyl acetate. Pellets were dehydrated through a graded series of ethanol and propylene oxide up to 100% propylene oxide and then incubated in a 1:1 mixture of propylene oxide and EMBed 812 (Electron Microscopy Sciences; 14120). Pellets were equilibrated in 100% EMBed 812 overnight, placed into fresh EMBed812 in flat embedding molds, and cured at 60°C. Ultrathin sections (∼70 nm) were cut, and grids were stained with uranyl acetate and lead citrate. Sections were imaged using a Thermo Fisher FEI Tecnai 12 transmission electron microscope, and micrographs were recorded using a Gatan OneView 16-megapixel camera.

### Data availability.

All data are included in the figures and supplementary information.
