# Supplementary material for: Discovery of a Diverse Set of Bacteria That Build Their Cell Walls without the Canonical Peptidoglycan Polymerase aPBP
Source: mBio. 2021 Jul 27;12(4):e01342-21. doi: 10.1128/mBio.01342-21 (PMC8406291; doi:10.1128/mBio.01342-21)
Supplement: FIG S1 [file mbio.01342-21-sf001.pdf]

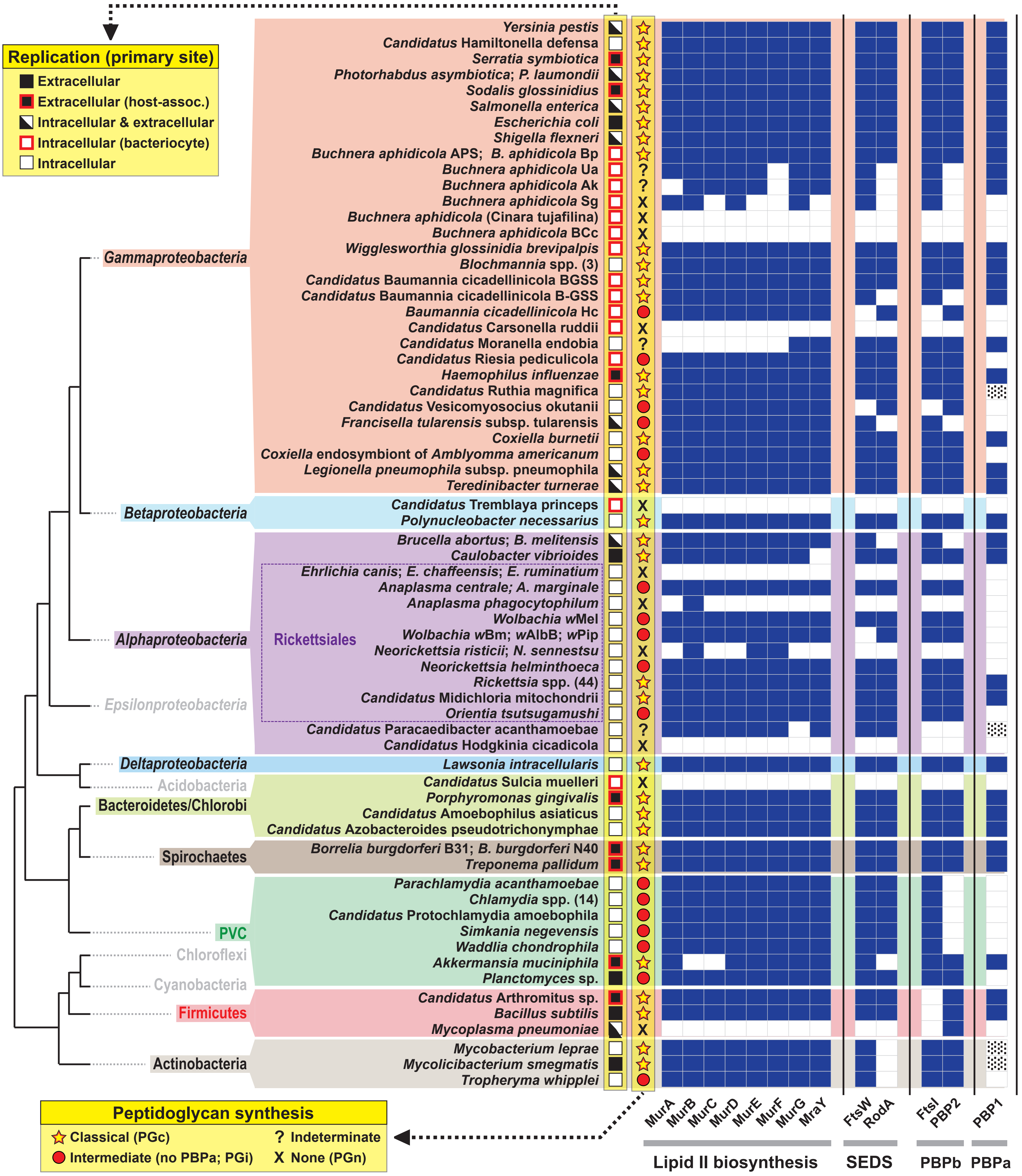

Supplementary Figure 1. The Class A PBP gene has been lost at least four times during evolution (extended version). Analysis of key genes in the peptidoglycan biosynthesis pathway showing their presence or absence in obligate intracellular, facultative intracellular, host-associated and free living bacteria across 9 phyla. The predicted peptidoglycan (PG) status is shown as well as the primary site of replication of the bacteria. Genes involved in lipid II biosynthesis, SEDS family, Class B PBPs and Class A PBPs were analyzed. The presence of a gene is shown by block color, the absence in white, and genes identified manually (not annotated as aPBP in KEGG) are shown with dashes. Bacterial organisms in Gammaproteobacteria (*Baumannia cicadellinicola* Hc, *Candidatus* Vesicomyosocius okutanii, *Francisella tularensis* subsp. tularensis, *Coxiella* endosymbiont of *Amblyomma americanum),* Alphaproteobacteria (*Anaplasma* spp., *Wolbachia* spp., *Orientia tsutsugamushi*), PVC (*Parachlamydia acanthamoebae, Chlamydia* spp., *Candidatus* Protochlamydia amoebophila, *Simkania negevensis, Waddlia chondrophila, Planctomyces* sp.) and Actinobacteria (*Tropheryma whipplei)* all lack Class A PBP homologs and are predicted to build intermediate PG-like structures (PGi). PVC = Planctomycetes, Verrucomicrobia and Chlamydiae, SEDS = shape, elongation, division and sporulation, PBP = peptidoglycan binding protein.
