# Supplementary material for: Discovery of a Diverse Set of Bacteria That Build Their Cell Walls without the Canonical Peptidoglycan Polymerase aPBP
Source: mBio. 2021 Jul 27;12(4):e01342-21. doi: 10.1128/mBio.01342-21 (PMC8406291; doi:10.1128/mBio.01342-21)
Supplement: FIG S2 [file mbio.01342-21-sf002.pdf]

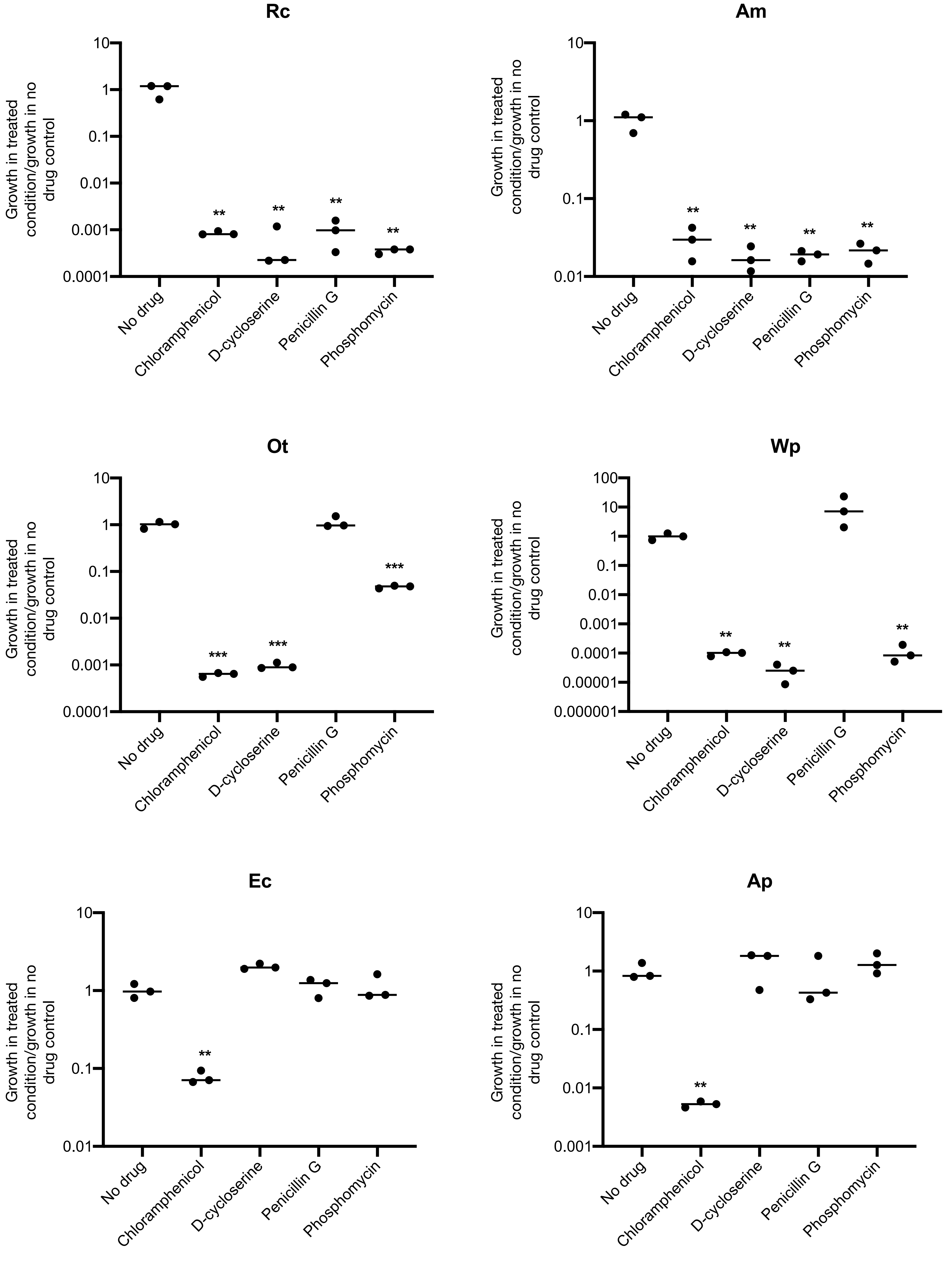

Supplementary Figure 2. Drug sensitivity assays. Bacterial copy number after 5 days (7 days for *Wolbachia)* growth in the presence or absence of drugs was quantified by qPCR. Growth in the presence of a drug was normalised by growth in the absence of drug within the same experiment. RC = *Rickettsia canadensis,* grown for 5 days in vero cells, AM = *Anaplasma marginale* grown for 5 days in vero cells, OT = *Orientia tsutsugamushi* grown for 5 days in L929 cells, WP = *Wolbachia pipientis* grown for 7 days in JW18 cells, EC = *Ehrlichia chaffeensis* grown for 5 days in DH82 cells, AP = *Analasma phagocytophilum* grown for 5 days in HL60 cells. Drug concentrations = 100 μg/ml chloramphenicol, 150 μg/ml penicillin G, 250 μg/ml d-cycloserine and 40 μg/ml phosphomycin. Statistical analysis between the untreated and drug treated groups was carried out using an unpaired t test with Graphpad Prism software. Individual values and median are shown. P values: * = $p \le 0.05$, ** = $p \le 0.01$, *** = $p \le 0.001$.
